# Glia Cell Morphology Analysis Using the Fiji GliaMorph Toolkit

**DOI:** 10.1002/cpz1.654

**Published:** 2023-01-23

**Authors:** Elisabeth Kugler, Eva‐Maria Breitenbach, Ryan MacDonald

**Affiliations:** ^1^ Institute of Ophthalmology University College London Greater London UK

**Keywords:** development, Fiji, glia, morphology, Müller glia, retina

## Abstract

Glial cells are the support cells of the nervous system. Glial cells typically have elaborate morphologies that facilitate close contacts with neighboring neurons, synapses, and the vasculature. In the retina, Müller glia (MG) are the principal glial cell type that supports neuronal function by providing a myriad of supportive functions via intricate cell morphologies and precise contacts. Thus, complex glial morphology is critical for glial function, but remains challenging to resolve at a sub‐cellular level or reproducibly quantify in complex tissues. To address this issue, we developed GliaMorph as a Fiji‐based macro toolkit that allows 3D glial cell morphology analysis in the developing and mature retina. As GliaMorph is implemented in a modular fashion, here we present guides to (a) setup of GliaMorph, (b) data understanding in 3D, including z‐axis intensity decay and signal‐to‐noise ratio, (c) pre‐processing data to enhance image quality, (d) performing and examining image segmentation, and (e) 3D quantification of MG features, including apicobasal texture analysis. To allow easier application, GliaMorph tools are supported with graphical user interfaces where appropriate, and example data are publicly available to facilitate adoption. Further, GliaMorph can be modified to meet users’ morphological analysis needs for other glial or neuronal shapes. Finally, this article provides users with an in‐depth understanding of data requirements and the workflow of GliaMorph. © 2023 The Authors. Current Protocols published by Wiley Periodicals LLC.

**Basic Protocol 1**: Download and installation of GliaMorph components including example data

**Basic Protocol 2**: Understanding data properties and quality 3D—essential for subsequent analysis and capturing data property issues early

**Basic Protocol 3**: Pre‐processing AiryScan microscopy data for analysis

**Alternate Protocol**: Pre‐processing confocal microscopy data for analysis

**Basic Protocol 4**: Segmentation of glial cells

**Basic Protocol 5**: 3D quantification of glial cell morphology

## INTRODUCTION

Cells in the central nervous system (CNS) have diverse and complex morphologies for their own and overall CNS function (Allen & Eroglu, 2017; Masland, 2012). Glial cells, the CNS support cells, have complex morphologies to connect neurons and blood vessels (Zonta et al., 2003), modulate neurotransmission, and impact neurogenesis (Argente‐Arizón, Guerra‐Cantera, Garcia‐Segura, Argente, & Chowen, 2017; Falk & Götz, 2017). As a part of the CNS, the retina is a tractable and accessible model to analyze glial cell shape during development. Müller glia (MG) are the principal retinal glial cell type, similar to astrocytes in the brain, with a unique morphology to support the function of several cell types by contacting and linking them functionally (Subirada et al., 2018). To fulfill these crucial functions, MGs have at least five domains that stretch radially with their apical domain from retinal photoreceptors to their basal endfoot in the inner limiting membrane (Wang et al., 2017). In addition to their intrinsic complex morphology, MG intercalate between cells in a so‐called tiled fashion, and thereby cover almost the entirety of the retinal space (MacDonald, Charlton‐Perkins, & Harris, 2017; Wang et al., 2017). Thus, glial shape is highly complex, and image‐based cell profiling can be used as a readout of MG function, maturity (MacDonald et al., 2017), and health (Halford et al., 2017). However, even though most MG studies rely on image‐based data, currently 3D image analysis tools quantitatively describing the stereotypic shape of MG, their cellular morphogenesis, or alterations thereof are lacking. So, even though we can use specific markers to look at MG (presence/absence) or stress protein expression, assigning meaningful measurements on glial morphology is challenging and not widely attempted. Thus, glial cell analysis is often based on visual or manual assessment, limiting reproducibility, throughput, and biological insights.

To study *in vivo* MG development, zebrafish have become a well‐established model, allowing insights into retina development and disease (Angueyra & Kindt, 2018; Gestri, Link, & Neuhauss, 2012; Malicki, Pooranachandran, Nikolaev, Fang, & Avanesov, 2016; Richardson, Tracey‐White, Webster, & Moosajee, 2017).

To address the current lack in terms of glia analysis workflows, we developed GliaMorph, a glia image analysis toolkit developed in the open‐source image analysis software Fiji (Schindelin et al., 2012) to analyze 3D glia morphology (Kugler et al., 2023). Briefly, GliaMorph covers:

- ***image pre‐processing***: to improve image quality by understanding data in 3D and performing deconvolution;
- ***semi‐automatic region‐of‐interest (ROI) selection***: to make images more comparable between samples and groups by semi‐automatic image rotation and 3D‐cropping;
- ***apicobasal intensity profile plots***: to assess texture and complexity, which can be applied to original, segmented, and skeletonized data;
- ***MG segmentation***: to binarize input data to background (non‐glia voxels) and foreground (glia voxels), which will be the foundation for extracting parameters such as volume and percentage volume coverage; and
- ***3D feature quantification***: which extracts (i) the surface to analyze surface, (ii) the Euclidean distance map to quantify thickness, and (iii) the skeleton to analyze skeleton length, number of junctions, and number of endpoints.

The design philosophy of GliaMorph is to provide readily applicable, open‐source, and easy‐to‐use tools for end users that can be adapted and expanded by users themselves as required. As GliaMorph was implemented in a modular fashion, tools can be combined depending on data and project needs. This also means that one could, for example, perform pre‐processing with alternate software before switching to GliaMorph, or, vice versa, use GliaMorph only for pre‐processing before performing data analysis elsewhere.

GliaMorph tools are implemented in Fiji, as the latter is open‐source, easy to use, and runs across platforms (Rueden & Eliceiri, 2017; Schindelin, Rueden, Hiner, & Eliceiri, 2015). Additionally, the ever‐growing community and documentation provide support and facilitate code adaptability if required. The implementation was done as macros, instead of a plugin, as macros are highly accessible to end users and easily adaptable if required. In the spirit of open science, we released the code on Github (https://github.com/ElisabethKugler/GliaMorph; doi: 10.5281/zenodo.6328234) and example data on Zenodo (https://zenodo.org/record/5747597).

**Figure 1:**
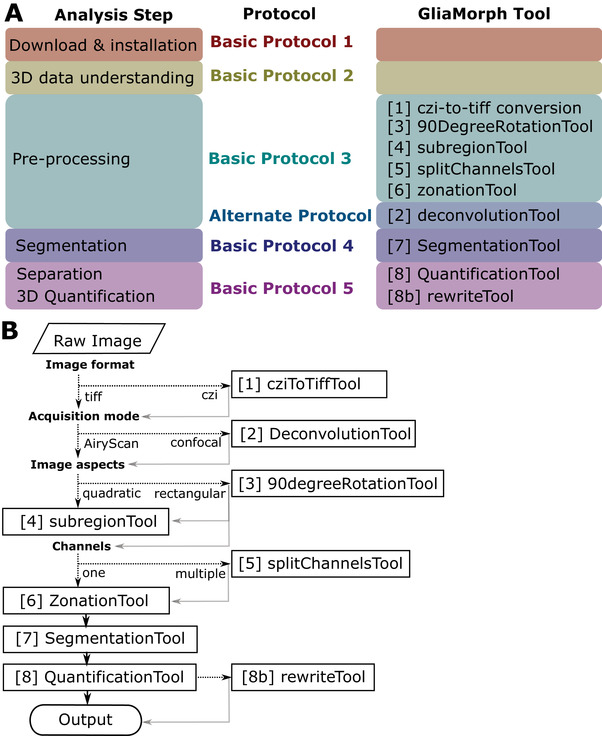
**(A)** Analysis steps covered by protocols in this manuscript, with the corresponding GliaMorph tool used at the respective steps. **(B)** Decision tree of GliaMorph analysis workflow.

Here we present step‐by‐step protocols that follow a typical image analysis workflow using GliaMorph (Fig. 1A). Basic Protocol 1 covers the download and installation of components and the example data used. Basic Protocol 2 describes how to examine data in 3D, as data understanding and integrity are the most crucial factors in biomedical image analysis. Basic Protocol 3 examines steps for AiryScan microscopy data pre‐processing, while the Alternate Protocol does this for pre‐processing of confocal microscopy data. Basic Protocol 4 presents approaches for the segmentation of glia cells; this step is pivotal and likely needs to be adapted for other visualization techniques, models, and tissues examined. Basic Protocol 5 describes the workflow toward 3D quantification of glial cell morphology and output data handling. For each protocol, example data are provided where appropriate.

Together, GliaMorph is a modular image analysis toolkit (Fig. 1B) that allows the quantification of glia morphologies and texture to understand fundamental cellular mechanisms in retinal tissues.

To support open science, please reference GliaMorph, this protocol, and the provided data accordingly.

## STRATEGIC PLANNING AND BASIC REQUIREMENTS

We do not discuss data acquisition and properties here, as this was addressed elsewhere (Kugler et al., 2023).

However, we wish to emphasize that high data quality is pivotal for meaningful data analysis outputs. Similarly, data derived from quantification should be used to inform back on acquisition settings to produce the most meaningful data analysis (Wait, Reiche, & Chew, 2020). GliaMorph macros are designed to work on 3D stacks of individual time points and iterate over images within given input folders (there is no need to open images; only a selection of the input folder is required). All tools require .tiff input images, with tool 1 (cziToTiffTool) converting .czi input data to .tiff if required. This can be easily adapted to other input formats such as .lsm. Similarly, we do not want to cover data organization in‐depth, but suggest the following three tips for navigating data (‐load) when using GliaMorph.

- Folder organization and naming should be done in a way that helps the user to easily find the desired experiment (e.g., YYYYMMDD_ExperimentDescription_ExperimentID). Unique and standardized folder naming is particularly important, as GliaMorph generally produces output folders within the input folder (i.e., unidirectional folder hierarchy; with input and output folders typically recorded in the Fiji log file). Do not use spaces or dots, but use camelCase or snake_case in file names—i.e., instead of 20211203 Rep1crossA. sample1 write 20211203_Rep1_crossA_sample1 (also see https://libguides.princeton.edu/c.php?g=102546&p=930626).
- Clustering files into experimental folders in order not to mix experimental conditions (e.g., folder 1: control, folder 2: treatment; with 8 images per folder, instead of 16 images in one folder). Especially when using GliaMorph for the first time, on a different system, or on differently acquired dataset, breaking files down into more manageable groups helps to identify potential issues or required optimization. Generally, when performing new analysis steps it is advised to run them first on a data subset or example data (we have included example data for all the relevant steps covered in this protocol).
- Lastly, reasonable and understandable file naming is key, e.g., do not name your files “1,2,…10”, but provide information at least on the experiment and sample ID. Similar to folder names, do not use spaces or dots, but use “camelCase” or “snake_case.”

To use GliaMorph, first Fiji needs to be installed. To do this, the reader is referred to https://imagej.net/Fiji/Downloads. Basic Protocol 1 will cover the steps for the Fiji installation, download of GliaMorph macros, and download of example data.

In terms of output files, the GliaMorph tools that produce data output documents produce .csv files that can be opened across platforms.

The required computational resources and computation time highly depend on the input image size (x,y,z dimensions, and color channels). We suggest always test run the workflow on a subset of images when using it for the first time. We examined various datasets on the following three systems, which allowed optimization of the code (Dell Precision 5820 Tower Windows 10 OS 128 GB; Workstation HP Z820 Windows 10 OS 64 GB; Mac Book Pro 2019 16 Gb). Should issues still arise, please look at the troubleshooting section; if this does not resolve the issues, please contact the authors.

Runtimes (hh:mm:ss) on Workstation HP Z820 Windows 10 OS 64 GB for an example workflow for a folder with eight two‐color channel images as input (3208 × 3208 × 50 for x,y,z, respectively) are as follows:

step1_cziToTiffTool: 00:07:47
step4_subregionTool: 00:05:41
step5_splitChannelsTool: 00:01:35
step6_zonationTool: 00:00:28 (run on one channel)
step7_SegmentationTool: 00:05:12 (run on one channel)
step8_QuantificationTool: 00:59:21 (run on one channel)

Total for this example: 01:20:04—again, the GliaMorph Toolkit is modular, and runtimes depend on input data properties, selected steps, and computational capacity. This does not include any subsequent data plotting or statistical analysis.

We recorded screencasts for the general GliaMorph workflow and shared them on YouTube: https://www.youtube.com/playlist?list=PLaAjG7r5mqQnmkPdktLJqbxotoRfyY72k.

## Basic Protocol 1 DOWNLOAD AND INSTALLATION OF GliaMorph COMPONENTS INCLUDING EXAMPLE DATA

GliaMorph was implemented as macros in Fiji, allowing it to run across platforms and be easily adaptable by end‐users (Rueden & Eliceiri, 2017; Schindelin et al., 2015). Again, we will not cover the installation of Fiji itself, as there is extensive information online (https://imagej.net/Fiji/Downloads).

This protocol aims to cover the setup of GliaMorph, including (a) which update sites are needed and how to do this, (b) the download of all required code, and (c) the download of the example data.

### Necessary Resources

#### Hardware

As described in the section Strategic Planning

#### Software

All GliaMorph macros can be found at https://github.com/ElisabethKugler/GliaMorph

#### Files

All example data can be downloaded at https://zenodo.org/record/5747597 [doi: 10.5281/zenodo.5747597]

### Fiji update sites and additional features

**Figure 2:**
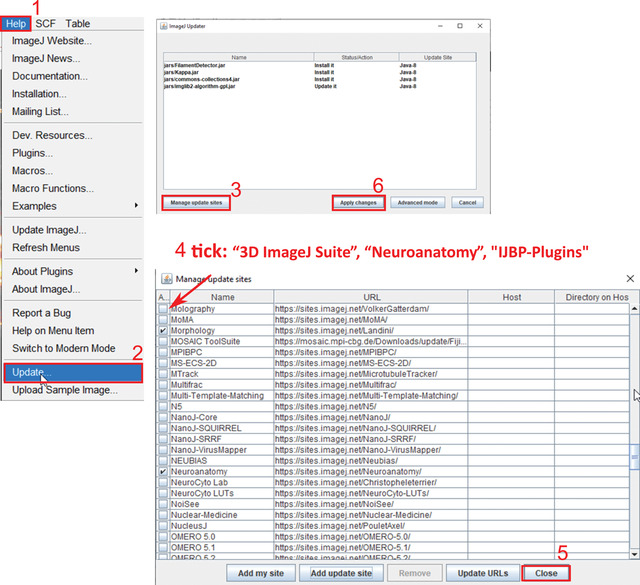
Steps to update Fiji sites.

1a To update the Fiji update sites, first open “Fiji > Help > Update > Manage update sites” (Fig. 2).

2a Select “3D ImageJ Suite,” “Neuroanatomy,” and “IJBP” plugins.
3a Click “Close.”
4a Click “Apply Changes.”
  These updates are required because the 3D ImageJ Suite is needed for 3D segmentation and processing (Ollion, Cochennec, Loll, Escudé, & Boudier, 2013) while the Neuroanatomy plugin is needed to summarize skeleton features.
5a Download two extensions to Fiji manually.
  Both are required for the “Alternate Protocol” point spread function (PSF) deconvolution.
  a. Extension 1: Diffraction PSF 3D to generate a theoretical PSF: details at https://www.optinav.info/Iterative‐Deconvolve‐3D.htm.
  • Download Diffraction_PSF_3D.class from https://github.com/ElisabethKugler/GliaMorph (found under “other”). Copy and paste this it into Fiji > Plugin folder > restart Fiji.
    Author: Bob Dougherty; Permission: 13.12.2021—via email between Bob Dougherty and Elisabeth Kugler; link: https://www.optinav.info/Diffraction‐PSF‐3D.htm; Licence: Copyright 2005, OptiNav, Inc. All rights reserved.
  • Check if "Plugins > Diffraction PSF 3D" is there.
  b. Extension 2: DeconvolutionLab2 for PSF deconvolution (Sage et al., 2017): follow the installation guide at http://bigwww.epfl.ch/deconvolution/deconvolutionlab2/.

### Code download

**Figure 3:**
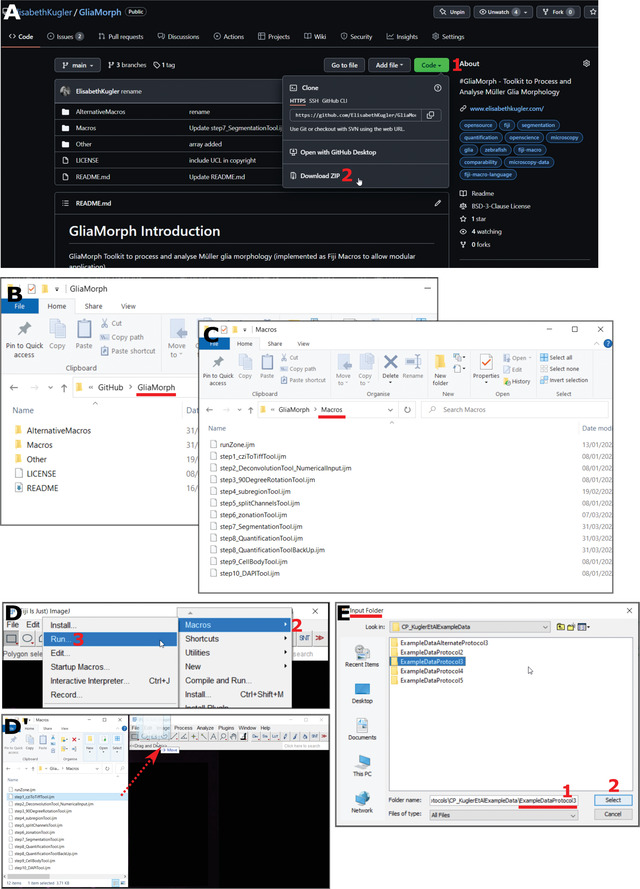
Steps to code. **(A)** Code download from https://github.com/ElisabethKugler/GliaMorph. **(B,C)** Downloaded code is in the folder “GliaMorph”; the macros are in the folder “Macros.” **(D,D’)** Steps to opening code either via Plugins > Macros > Run, or “Drag and Drop” from folder. **(E)** The macros are designed to run on all tiff files in folders; thus each macro prompts for the selection of an “input folder.”

1b Go to https://github.com/ElisabethKugler/GliaMorph and download the macros as a .zip file (Fig. 3A).

2b Extract the .zip file in the folder where you downloaded it.
  You can move the folder containing the macros “GliaMorph” to another location, e.g., internal or external drive.
  In the folder, you will find subfolders. The macros (.ijm files) used in this protocol (Fig. 3B,C) are in the Macros subfolder.
3b Open macros:
  - Option 1: Fiji > Plugins > Macros > Run (Fig. 3D)
  For more information visit https://imagej.net/scripting/macro and https://imagej.nih.gov/ij/developer/macro/macros.html.
  - Option 2: Open Fiji, then drag and drop the macro (.ijm) from the folder GliaMorph>Macros into Fiji (Fig. 3D).
    All macros are designed to iterate over all .tiff files of the selected input folder.
    They are called stepX_name.ijm, where X denotes a number, name is a descriptive name, and .ijm is the macro file ending.
    Where appropriate, a graphical user interface (GUI) will appear with options (for example number of channels or image output size).
    All macros will automatically create output folders in the input folder with output data that are automatically named (i.e., files are overwritten if the same macro is run iteratively on the same folder).

### Example data download

We provide example data for each of the following protocols, including input data, output data, results files, and descriptions as appropriate.

Download data from https://zenodo.org/record/5747597 [DOI: 10.5281/zenodo.5747597].

Data are named ExampleDataProtocolX where X denotes the number of the protocol, respectively.

## Basic Protocol 2 UNDERSTANDING DATA PROPERTIES AND QUALITY 3D—ESSENTIAL FOR SUBSEQUENT ANALYSIS AND CAPTURING DATA PROPERTY ISSUES EARLY

As your data are 3D, it is important to understand and examine them in 3D. This protocol is suggested to be performed for each new dataset and/or when imaging parameters are changed. Understanding your data quality will allow you to improve, for example, sample preparation (e.g., tissue penetration of antibodies) or image acquisition (e.g., laser power settings). The outcomes of this protocol are however not needed for GliaMorph *per se*, and are here solely to guide the user on data understanding and data quality; i.e., if your segmentation is unsatisfactory, this is most likely due to data quality, etc.

GliaMorph performs data analysis in 3D; therefore end users must consider all three image dimensions equally. This is particularly important because images are usually examined as stacks with a slider when only one slice is visible at a time, but data are 3D; thus slices should be examined in the context of their neighbors. Similarly, in confocal imaging, the penetration decreases over the z‐axis, leading to uneven data quality in the third dimension, meaning that typically the “top” of the stack will have a higher quality than the “bottom” (Tröger et al., 2020). We previously examined cell properties and data acquisition considerations in 3D (Kugler et al., 2023). Here, we give you pointers on how to check data integrity visually by examining voxel properties and using the Fiji 3D Viewer (Schmid, Schindelin, Cardona, Longair, & Heisenberg, 2010), as well as how to assess image quality quantitatively using signal‐to‐noise (SNR) or contrast‐to‐noise ratio (CNR). To some readers, this protocol might sound intuitive, but we have encountered various instances where quantification outcomes were wrong due to, for example, altered voxel properties (typically happening during image export, import, or saving).

### Examining voxel properties

**Figure 4:**
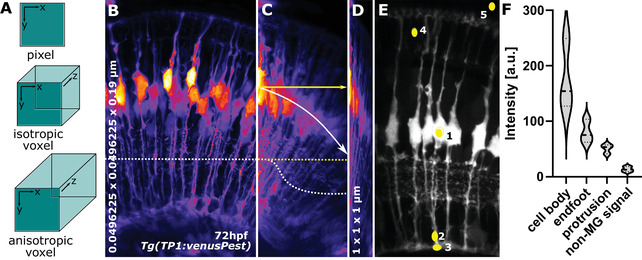
**(A)** Pixel and voxel properties. **(B)** MIP of MG at 72hpf with an isotropic resolution of 0.0496225 × 0.0496225 × 0.19 μm (x,y,z, respectively). The dotted line indicates homogenous signal distribution across the x‐axis. **(C)** Image **(B)** but horizontally resliced, showing signal decay across z‐axis (white dotted line, yellow dotted line would be the ideal) and displaying image angle (white arrow, with yellow, are being the ideal). **(D)** Image **(B)** but with voxel properties being artificially altered to 1 × 1 × 1 μm. **(E)** ROI for CNR and SNR quantification.

Voxels are 3D cubes (Fig. 4A), which needs to be considered when performing 3D processing and analysis steps, as many calculations depend on this. Additionally, due to resolution limits, voxels are typically longer in (z) than they are wide in (x,y), meaning voxels are shaped like a rectangular cuboid, making them “anisotropic.”

1a Open Fiji and open TP1venusPest_72hpf_originalResolution.tiff from the folder ExampleDataProtocol2 in Fiji.
  Typically, data are 3D stacks of 2D slices in a .tiff format or a specific microscopy format, such as Zeiss .czi. If you are unsure on how to open these, the user is referred to the following pages from Fiji (https://imagej.net › mbf › importing_image_files) or the Bioformats library (Linkert et al., 2010).
2a Click Fiji Image > Properties.
  This will provide information on channels, slices, frames, and voxel x,y,z dimensions, as well as unit (e.g., micrometer or px). You do not need to note this information, as it is only for your understanding.
  Characteristic issues that we have seen at this step are (i) slices are interchanged with frames (e.g., 3D becomes 2D+time) or (ii) that correct voxel dimensions are lost [e.g., voxels become falsely isotropic (Fig. 4B‐D].
  At the end of this step, you should understand the shape of your voxels. Example (a), if your z‐stack interval is 5 µm while your x,y size is 0.03 µm—do you think it is meaningful to apply a filter that things your voxels are a cube? Example (b), if your structure of interest is 1 µm—what is the ideal sampling frequency to capture this?
  While this step might seem intuitive, in our experience, suboptimal voxel properties and data acquisition are the main cause for meaningless data quantification, i.e., rubbish in = rubbish out.

### Visualizing data using the Fiji 3D viewer

1b Keep the above image open.
2b Open it with the Fiji 3D Viewer by selecting Plugins > 3D Viewer.
3b Do not change the default settings.
4b Select “OK.”
  The web page https://imagej.net/3D_Viewer contains very useful demos. If you have never used the 3D viewer before, we suggest you examine this link first (Schmid et al., 2010).
5b Examine the data in 3D by rotating and zooming (also see https://www.youtube.com/watch?v=Hb3tDVJ4KXU from approximately 46 min).
  The 3D rendering might take a few minutes to finish. You can leave this running in the background and assess the outcomes later.
  At this stage, data quality can be visually assessed, e.g., pay attention to the following. (i) Do the data looked squished or stretched (see “voxel properties” above); (ii) are the data at a significant angle, rendering them challenging to quantify (Fig. 4C); (iii) do the data suffer from significant z‐axis signal loss, requiring them to be specifically pre‐processed (Fig. 4C); or (iv), what does the point‐spread‐function (PSF) look like.
  Again, this step itself is not needed for GliaMorph, but is crucial to inform back on image acquisition improvements and the requirements that must be met for further analysis.

### CNR and SNR

CNR and SNR are assessments of image quality that can be used to study how individual processing steps change image quality, how different visualization techniques perform against each other, or differences within cells. Here we show an example, examining MG cell bodies, protrusions, and endfeet. However, for some studies, it might be sufficient to examine only one of these.

**8‐bit** conversion before the following steps can be useful for image comparability ((Image>type>8 bit).

### Region of interest (ROI) selection example

1c Open one 3D tiff stack.
  This can be one color or multiple, but we would suggest to first start with individual channels as to not confuse ourselves. For example: Fiji > Image > Color > Split Channels.
2c Once the stack is opened > move slider to a position in the stack where a full MG can be seen.
3c Select circular ROI in the cells of interest [e.g., for MG: cell body (5 × 5 μm), protrusion (2 × 5 μm), and/or endfoot (5 × 3 μm); Figure 4E ROI 1‐3; example ROIset is in the folder ExampleDataProtocol2]. Select the oval sign on the toolbar and then draw the circle on the region of interest.
4c Measure signal by selecting Analyze > Histogram.
  This will bring up a window asking “Include all XXX images?”, where XXX denotes the number of open images. Select “no,” which means you measure the intensity only in the selected slice.
5c Write down mean signal “***Mean***.”
  See a similar approach but for blood vessels at https://www.youtube.com/watch?v=Hb3tDVJ4KXU from approximately 56 min. This will be referred to as “mean inside,” as it is measured within cells.
6c In the histogram window, select the button “live,” meaning the values will automatically change when the ROI is moved.
7c Select the image and move to background within the eye (Fig. 4E, ROI 4).
8c Measure mean signal “***Mean***.”
  This will be referred to as “mean outside,” as it is outside the cell, but within the tissue.
9c Move to background outside the eye (Fig. 4E ROI 5).
10c Measure the standard deviation “***StdDev***.”

### Saving individual ROIs

After creating the ROIs and measuring the respective intensity values, the ROIs need to be saved, so you can later use the same ROIs to measure the impact of image processing.

1d After drawing the ROI and measuring the values (see above), add each ROI‐to‐ROI manager:
2d “Edit > Selection > Add to Manager”
3d Select the image instead of histogram.
4d Click “Add (t)” in ROI manager.
5d In the ROI manager: More > Save (save ROI with a meaningful name, e.g. *ROI_cellBody_sample1*).

### Calculate CNR or SNR

See example calculations in the file ExampleCNR in folder ExampleDataProtocol2 (Fig. 4F; see example Excel file ExampleCNR.xlsx).
Formulas:
(1) $$CNR=\;\frac{\mu_{s}\;-\;\mu_{ns}}{\sigma_{bg}}$$
(2) $$SNR=\;\mu_{s}\;-\;\mu_{ns}$$
where **µ_s_** is the mean signal, **µ_ns_** is the mean non‐signal, and **σ_bg_** is the standard deviation of the background.
  For example, in ExampleCNR.xlsx **µ_s_** are ROI_cellBody, ROI2_protrusion, ROI3_endfoot; **µ_ns_** is ROI2_nonSignal; and **σ_bg_** is ROI5_outsideRetina.

### Analyzing the impact of processing (optional)

This would typically be done after pre‐processing steps, such as PSF deconvolution.

1e Process the above images (e.g., to try it you could use a median filter, which is a filter that allows you to smooth the data and is often used to remove image speckles; you can find it under “Process > Filters > Median 3D.” You can just test a scale of 5 × 5 × 5 for this exercise).
2e Perform measurements again using the original ROIs.
3e Recalculate CNR and SNR.
4e Assess whether filtering increased or decreased CNR and/or SNR.
  At the end of this protocol, you will understand how your data are composed by voxels, how to assess 3D image properties using a 3D viewer, and how to quantitatively assess image quality using SNR/CNR measurements. While these steps seem simple on their own, performing them after processing steps will give you an idea about how data are changed using selecting processing steps.

## Basic Protocol 3 PRE‐PROCESSING AiryScan MICROSCOPY DATA FOR ANALYSIS

In this protocol, we cover the image preprocessing of data acquired with AiryScan microscopy. As AiryScan microscopy and processing are specific to the microscope with which the data were acquired, the reader is referred to perform AiryScan processing [non‐iterative linear Wiener deconvolution algorithm (Huff, 2015; Zeiss, 2019) as indicated by their microscope manual].

Below, the following 4 operations will be presented:

- File format conversion: e.g., .czi to .tiff using Bioformats (Linkert et al., 2010).
- Increasing image comparability by image cropping and orienting.
- Splitting of multi‐channel images.
- Data analysis using a 1D‐vector.

### (a) File format conversion using cziToTiffTool

This tool is to convert .czi to .tiff to allow subsequent data processing in .tiff format. It works on single and multi‐color images.

1a Drag and drop step1_cziToTiffTool.ijm from the folder GliaMorph > Macros into Fiji.
2a Click “Run.”
3a Select folder with folder with input files (i.e., folder with .czi files).
4a Click “OK.”
5a Check the generated output folder “tiff” in the input folder with stacks and MIPs.
  Example Data: In the folder ExampleDataProtocol3 you will find three .czi files and the output folder “tiff.”
  Before running the macro on the example data (do not do this for real analysis workflows), we would recommend you renaming the “tiff” folder to something like “tiffExample”; otherwise, the folder and everything in it will be overwritten (this holds true for all the subsequent steps).
  Should you have accidentally overwritten the “tiff” folder, you can always re‐download the example data folders and start afresh.

### (b) Establishing data comparability using the subregionTool

**Figure 5:**
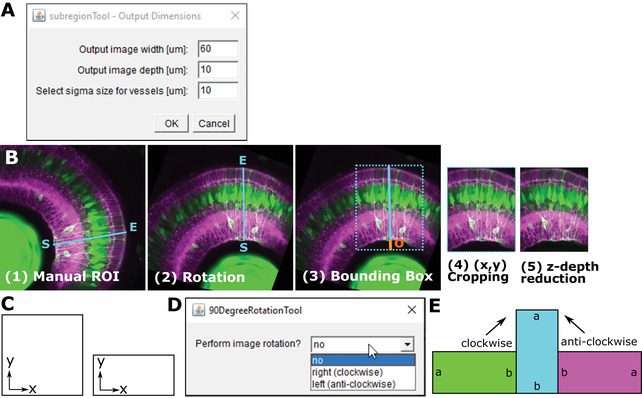
**(A)** GUI of the rotationTool Macro, including parameters for output image width, image depth, and sigma. **(B)** Overview of rotationTool processing. **(C)** Images can be square or rectangular in x‐y. **(D)** GUI for the 90DegreeRotationTool. **(E)** Images are rotated clockwise or anti‐clockwise with the 90DegreeRotationTool.

This step aims to orient all stacks in the same direction via x‐y rotation and making stacks the same size (x,y) and depth (z) by cropping (Fig. 5A,B). The default parameters are based on manual measurements (Kugler et al., 2023) and can be adjusted according to data needs (for non‐square data, see 90DegreeRotationTool below).

### Options of the GUI (Fig. 5A)

- Width of the output stack in μm. Default output image width = 60 μm, as we found this to include ∼5‐6 MG laterally (code variable “xySize”). If your stacks tend to be wider, you can make the output wider. While this increases the information, this ultimately increases computation time.
- Depth of the output stack in μm. Default output image depth = 10 μm, as we found this to include ∼1‐2 MG in depth (code variable “zDepth”). If your stack is larger, you can increase this. We suggest 10 μm as a minimum, due to MG thickness/properties. When going over 10 μm, we suggest to go back to Basic Protocol 2 to assess whether this is meaningful, or whether z‐axis signal decay is too severe.
- Addition of length to the ROI to account for retinal curvature, and or to include blood vessels that underlie the endfeet. Default sigma size = 10 μm. When setting this to 0 μm, lateral endfeet tend to be cut‐off due to retinal curvature. Depending on your samples you can increase/decrease this value. Generally, this adds only minimal computation time when added, so we suggest to keep this. Input data are in the folder with .tiff files (e.g., after step1) and a RoiSetLine.zip, which is in the same folder as the .tiffs. See below how to create this.

1b Open MIPs (.tiff format, but MIPs can be multi‐channel) of each image individually to place ROI.
*IMPORTANT NOTE*: Open images in the order they appear in the folder (can be any bit‐format, e.g., 8‐bit or 16‐bit).
2b Place a line ROI starting from the inner part of the retina and extending outwards on each image.
3b *IMPORTANT NOTE*: Place ROI at the position with the best signal and data; this will be the center of the created output. Click “Straight” on tool bar.
4b Add each ROI to the ROI manager [Edit > Selection > Add to Manager > Add (t)].
  If you have just one image, then add the ROI line twice to the manager.
5b Save all ROIs as RoiSetLine.zip. Make sure to rename the default RoiSet.zip to RoiSetLine.zip (this was implemented to avoid overwriting of ROI folders).
*IMPORTANT NOTE*: Save this ROI folder into the “tiff” folder (not in the MIP folder) and run on the 3D “tiff.”
6b Close everything.
7b Drag and drop step4_subregionTool.ijm from the folder “GliaMorph>Macros” into Fiji.
8b Click “Run.”
9b Select folder with folder with input files (i.e., folder “tiff” from Basic Protocol 2 or a folder containing .tiff files).
10b Change the width, depth, or sigma if desired.
  Even though the ROIs were drawn on the MIPs, the SubregionTool will run on the 3D stacks.
11b Click “OK.”
12b Check the generated output folder “zDir,” containing images rotated along the x‐y axis and reduced in the z‐axis direction.
**Example Data**: In the folder ExampleDataProtocol3\tiff\90DegreeRotated you will find input data and the RoiSetLine.zip. The output data are in the folder “zDir.”
**90DegreeRotationTool**: Typically, acquired images are in a square format, meaning x and y have the same dimension (Fig. 5C‐E). In some cases, images might be acquired in a more rectangular fashion. If this is the case, “90DegreeRotationTool” has to be applied before the “subregionTool.”
  This step is optionally applied before the rotationTool, to be applied to images that are not quadratic (e.g., 1920 × 1920 in x and y, respectively) but rectangular (e.g., 512 × 1920 in x and y, respectively).

### Options

- No—application will exit;
- right (clockwise) – images will be rotated 90 degrees in clockwise orientation;
- left (anti‐clockwise)—images will be rotated 90 degrees in anti‐clockwise orientation.

- Drag and drop step3_90DegreeRotationTool.ijm from the folder “GliaMorph>Macros” into Fiji.
- Click “Run.”
- Select folder with folder with input files (i.e., folder “tiff” from Basic Protocol 2 or a folder containing .tiff files).
- Click “OK.”
- Check the output folder “90DegreeRotated” in the input folder with stacks and MIPs.
**Example Data**: In the folder ExampleDataProtocol3/tiff you will find the input data, and the output data in ExampleDataProtocol3/tiff/90DegreeRotated that were rotated clockwise.
  In some cases, images might be in very large stacks where the cells of interest are not located at the start of the stack, but more centrally (for example to visualize clones). We here suggest drawing 3D line ROIs within the stack.

- Open stack (caveat: before we used the MIPs .tiff).
- Select plane of interest.
- Draw line ROI as above along cells of interest.
- Save ROISetLine.zip as above.
- Drag and drop step4_subregionToolWithinStack.ijm from the folder “GliaMorph>Macros” into Fiji.
- Click “Run.”
- Select folder with folder with input files (i.e., folder “tiff” from Basic Protocol 2 or a folder containing .tiff files).
- Click “OK.”
  This will rotate and perform x,y‐reduction as above, but additionally extract the sub‐stack within the stack (the drawn ROI will be at the center, given there are enough slices above and below—if not, then the stack will be started in a position where it fits).

### (c) Separating multi‐channel images with the splitChannelsTool

This step automatically splits channels of all images in a folder using the Fiji split channels option and saves them in separate folders.

**Figure 6:**
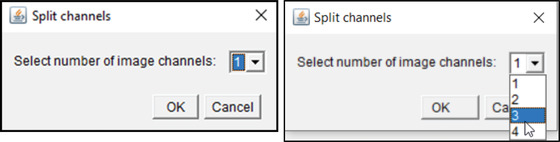
GUI of the splitChannelsTool.

***Options***: Select number of channels (channels 1‐4; Fig. 6).

1c Drag and drop step5_splitChannelsTool.ijm from the folder “GliaMorph>Macros” into Fiji.
2c Click “Run.”
3c Select number of channels from the drop‐down menu.
4c Select folder with folder with input files (i.e., folder “zDir” from Basic Protocol 3).
5c Click “OK.”
6c Check output folder “XCDir” (“X” indicating the channel number) in the input folder with stacks named XC‐name (“X” indicating the channel and “name” being the original filename).
**Example Data**: In the folder ExampleDataProtocol3\tiff\90DegreeRotated\zDir you will find the input data, and the output folders 1CDir, 2CDir, and 3CDir.

### (d) Examining data integrity and texture with the ZonationTool

**Figure 7:**
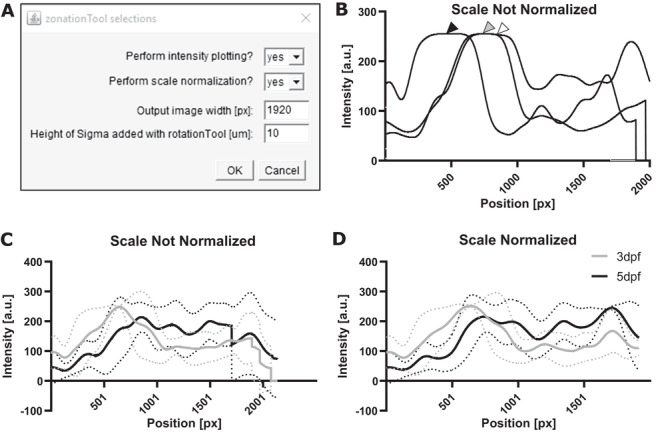
ZonationTool overview. **(A)** GUI of the zonationTool. **(B)** Example of plots from three different images, where gray and white arrowhead indicate overlap, and the black arrowhead indicates that cell bodies in this sample are not aligned with the others. **(C,D)** Same data plotted without and with scale normalization, respectively.

The zonationTool produces 1D vector from 3D stack to derive apical‐to‐basal intensity plots. These plots indicate the relative position of MG sub‐domains along the apicobasal axis. Plotting the derived intensities allows insights into data integrity, similarity, and texture, e.g., “Are the SubregionTool outputs satisfactory?” or “Are the examined data acceptably age‐matched?” (Fig. 7A,B).

### Options (Fig. 7)

- *Perform intensity plotting*: If “no” is selected nothing happens.
- *Perform scale normalization*:
  ○ “yes”—output data will be scaled to the same size (selected in the next box);
  ○ “no”—output data are the size of the input (Fig. 7C,D).
- *Output image width*: Image scale for rescaling to make images comparable.

*Height of sigma*: To measure retina height (sigma selected in rotationTool—i.e., if you selected 10 µm before, add 10 µm here).

***For the input folder, select the folder with tiff files after the subregionTool (it is important that these are the same size and comparable—i.e., it will not work on original input tiffs); for multi‐channel images apply splitChannelsTool first and apply to images from different channels individually***.

1e Drag and drop step6_zonationTool.ijm from the folder “GliaMorph>Macros” into Fiji.
2e Click “Run.”
3e Select steps and parameters.
4e Select folder with folder with input files [for single channel images—zDir; for multichannel images—the respective folder (1C/2C/3C/4C) inside the zDir folder).
5e Click “OK.”
6e Check the output folder “ZonationTool” in the input folder containing 1D vectors with LUT fire, a .csv with apical‐to‐basal intensity profiles (“ZonationToolProfiles”) and a .csv file with measurements (“ZonationResults”).
***Example Data***: The input data are in the folder ExampleDataProtocol3\tiff\90DegreeRotated\zDir\2CDir, while the example output data can be found in ExampleDataProtocol3\tiff\90DegreeRotated\zDir\2CDir\ZonationTool.
  After this, continue with Basic Protocol 4.

## PRE‐PROCESSING CONFOCAL MICROSCOPY DATA FOR ANALYSIS

This protocol sequence is like the above; however, the input data are acquired with conventional confocal microscopy rather than AiryScan microscopy, meaning that a separate deconvolution step is required. This is due to the fact that when imaging, convolution of light in 3D produces a so called point spread function (PSF; see Gennaro & Geoff, 2010; or https://bitesizebio.com/22166/a‐beginners‐guide‐to‐the‐point‐spread‐function‐2/), which means that, for example, originally spherical objects appear elliptical—using computational deconvolution based on image acquisition knowledge the object can be deconvolved to the original spherical shape.

**Figure 8:**
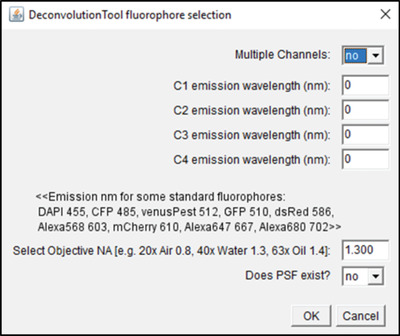
GUI of the DeconvolutionTool.

Therefore, PSF deconvolution is applied to improve data quality (Shaw & Rawlins, 1991). The DeconvolutionTool (Fig. 8) supports PSF deconvolution at different wavelengths using the DeconvolutionLab2 Plugin (Sage et al., 2017; http://bigwww.epfl.ch/deconvolution/deconvolutionlab2/ which was downloaded in Basic Protocol 1, “b” steps for code download) and supports the use of existing or non‐existing PSF files (Fig. 8). In the case of non‐existing PSF files, it is modeled using analytical derivation based on Fraunhofer diffraction using the “Diffraction PSF 3D” Plugin (https://imagej.net/Deconvolution; https://www.optinav.info/Diffraction‐PSF‐3D.htm’ again, these were downloaded in Basic Protocol 1, “b” steps for code download).

If you have access to another deconvolution tool, you can proceed to the next step, i.e., image segmentation, and use your.tiff files that were deconvolved elsewhere as input.

Here we use PSF deconvolution using Richardson and Lucy with 1 iteration (DeconvolutionLab2) to be applied to confocal images (redundant for AiryScan processed data); the tool works with and without existing PSF.

Options:

***Multiple channels***: Specify the number of channels in the input images. The default will be “no,” assuming that there is only one channel as input. Select “yes” if there are multiple channels as input.
***C1‐C4***: Select which fluorophores were imaged: e.g., if your first channel is GFP, then you need to write 510 into the first box; if your second channels is dsRed, then you need to write 586 into the second box.
***Select objective NA***: Only required if PSF does not exist, meaning it will produce a theoretical PSF; specify based on the objective numerical aperture.
***Does PSF exist?***: If PSF does not exist, it will generate a theoretical PSF using Diffraction PSF 3D. If PSF exists (experimental or theoretical), the macro will prompt the user to select a PSF file for each channel.

***For the input, select the folder with .tiff files acquired in confocal mode. We recommend that you apply the SubregionTool first, as this will make the data smaller and therefore the deconvolution step quicker. Caveat: deconvolution is computationally intensive, so it might not be suitable for low‐capacity machines***.

1 Drag and drop step2_DeconvolutionTool.ijm from the folder “GliaMorph>Macros” into Fiji.
2 Click “Run.”
3 Select options.
4 Select folder with folder with input files (for single channel images—zDir; for multichannel images—the respective folder (1C/2C/3C/4C) inside the zDir folder).
5 Click “OK.”
  As this step is computationally very intensive, we advise it to be run on data that are reduced in size by step4_subregionTool.ijm. Once the tool has finished, all windows can be closed.
6 Check the Output:
  - ***ChannDir***—directory with images of individual channels.
  - ***PSFDir***—directory with theoretically produced PSF files.
  - ***DeconvDir***—directory with the deconvolved images—these are the data that should be used for subsequent data analysis steps.
***Example Data***: The folder ExampleDataAlternateProtocol3 contains input files and output folders created with PSF deconvolution, using theoretically created PSF files.
  For confocal data, we suggest applying the SubregionTool before the deconvolutionTool, as PSF deconvolution is computationally intense and reduced data size reduces the time needed for computation.

## Basic Protocol 4 SEGMENTATION OF GLIAL CELLS

Subsequent to image understanding and pre‐processing, image segmentation is applied to extract cells from images by binarization (i.e., foreground/cells = 1; background = 0). Computationally, this is very challenging, as glia have complex morphologies (MacDonald et al., 2017; Wang et al., 2017) and suffer visualization heterogeneity (Halford et al., 2017; Escartin et al., 2021), making them more difficult to segment than, for example, cells with a round shape and homogenous signal distribution.

As image segmentation depends on a myriad of factors, we can here only provide a brief overview and the reader is referred to publications on segmentation factors and assessment (Bolón‐Canedo & Remeseiro, 2020; Jeevitha, Iyswariya, RamKumar, Basha, & Kumar, 2020; Udupa et al., 2006).

Independent of segmentation approach and desired analysis, for segmentation to be meaningful and automatable, images need to be comparable in quality and properties. Often this can be achieved by visual grading [e.g., are images looking the same throughout the dataset, are they roughly the same size, are there any artifacts (Koho, Fazeli, Eriksson, & Hänninen, 2016)] and measurements such as CNR (see Basic Protocol 1).

In our experience, researchers often use the first segmentation approach that looks suitable for their data. However, we find that this often means sub‐optimal segmentation results unless there is further assessment, optimization, or validation. Therefore, even if data segmentation and quantification are presented in manuscripts, they must be assessed with caution.

Here we examine how to examine segmentation workflows, with the caveat that project‐specific segmentation is likely to require optimization.

### Examining segmentation for suitability

1a *Manual testing*: Before the automation of any segmentation workflows, it is advised to examine different segmentation methods, parameters, and settings manually to gauge ranges. See basic introduction on preprocessing, segmentation, and analysis at https://imagej.net/imaging/segmentation. Importantly, making notes or using the recording tool (Plugins > Macros > Record) helps to achieve a structured approach in examining segmentation approaches.
2a SegmentationTest.ijm: Macro that allows testing of six commonly used segmentation approaches, namely 3D simple segmentation using the 3D Segmentation plugin (Ollion et al., 2013), Hysteresis (histogram‐derived) using the 3D Segmentation plugin (Ollion et al., 2013), Otsu thresholding (Otsu, 1979), Moments (Tsai, 1995), Percentile (Doyle, 1962), and Maximum Entropy (Kapur, Sahoo, & Wong, 1985).
3a Do SegmentationTest.ijm with pre‐processing.
  Tests the above six thresholding methods, but with image smoothing and background removal (uncomment line 40 in the macro).
4a SegmentationTest.ijm with PSF deconvolution:
  As in step 3a but with prior PSF deconvolution (see Alternate Protocol).
5a *S* egmentationTest.ijm with pre‐processing with PSF deconvolution:
  Combination of step 3a and step 4a.

***For the input folder, select the folder with .tiff files (if you applied the SubregionTool to AiryScan data, then use the folder “zDir” as input; if you performed deconvolution, then either use “zDir” or “DeconvDir” depending on which you performed last)***.

**Figure 9:**
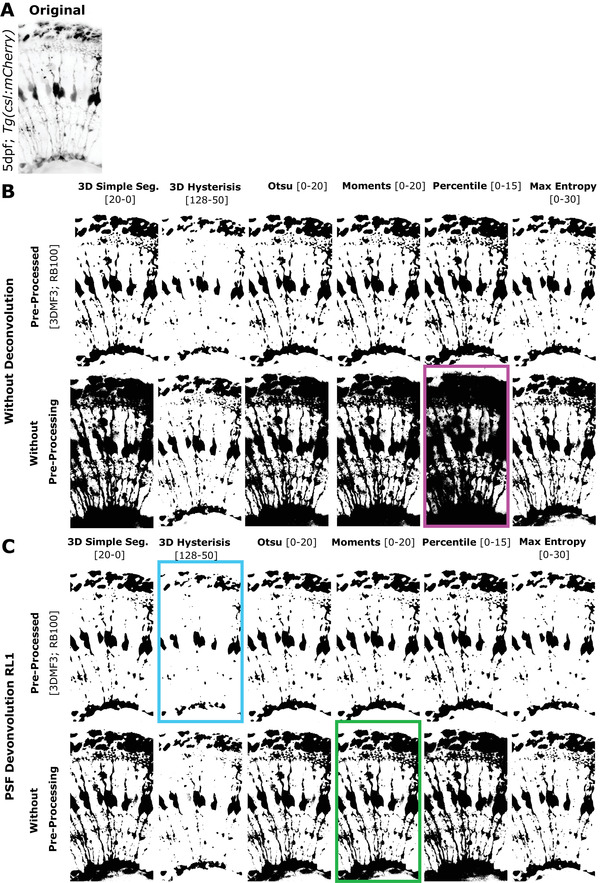
Preliminary tests for segmentation. **(A)** Original. **(B)** Segmentation methods tested on images without PSF deconvolution and with (upper panel) or without pre‐processing (lower panel). **(C)** Segmentation methods tested on images with PSF Richardson‐Lucy (RL) (1 iteration) deconvolution and with (upper panel) or without pre‐processing (lower panel; MF – Median filter, RB – Rolling Ball algorithm). ***Examples***: Cyan box: Under‐segmentation, magenta box: over‐segmentation, green box: Suitable segmentation for further fine‐tuning.

1b Drag and drop SegmentationTest.ijm from the folder “GliaMorph>AlternativeMacros” into Fiji.
2b Click “Run.”
3b Select options.
4b Select folder with folder with input files [depending on your previous steps you may select for single‐channel images—zDir; for multichannel images—the respective folder (1C/2C/3C/4C) inside the zDir folder. If you performed deconvolution, select the respective DeconvDir).
5b Click “OK.”
***Output***: Segmented tiff stacks and folder containing MIPs.
  Following the testing of different processing and segmentation workflows, comparing the results can be used to assess their suitability. Directly comparing segmentation workflows enables visual assessment, showing things such as under‐ (Fig. 9, cyan box) or over‐segmentation (Fig. 9, magenta box).
  Comparing segmentation outcomes can also be used to fine‐tune the parameters of the respective segmentation approaches (Fig. 9, green box).
  Segmentation is a multi‐step process, often containing preprocessing and segmentation steps (see macro code SegmentationTest.ijm line 40 and 41). Testing various parameters, such as filtering (line 54) or background removal (line 57), will give you an idea on how your data respond to individual steps.
  However, in‐depth explanations on how to optimize and validate segmentation goes beyond this protocol, and the interested reader is referred to Kugler, Rampun, Chico, & Armitage (2020).
  After visual assessment, validation of segmentation workflows including aspects such as accuracy, robustness, or speed is required (Padfield & Ross, 2009; Kugler et al., 2020). This is of particular importance, as only accurate segmentation will deliver biologically relevant information. In addition, the more sensitive a, segmentation approach, the fewer samples are typically needed to extract biological differences between examined groups. However, while extensive segmentation validation and testing are applied in medical image analysis, this is still often lacking in biomedical image analysis, and in our experience, segmentation approaches are often not optimized or validated and are therefore to be treated with caution.
***Example Data***: The folder “ExampleDataProtocol4” contains input and output files.
  GliaMorph contains two main segmentation approaches: (a) CytosolSegmentation.ijm that was optimized for Tg(TP1bglob:VenusPest)^s940^ (Ninov, Borius, & Stainier, 2012) and Tg(CSL:mCherry)^jh11^ (also known as Tg(Tp1bglob:hmgb1‐mCherry)jh11 (Parsons et al., 2009), and (b) MembraneSegmentation.ijm that was optimized for Tg(TP1:CAAX‐eGFP)^u911^.

***For the input folder, select the folder with .tiff files (if you applied the SubregionTool to AiryScan data, then use the folder “zDir” as input; if you performed deconvolution, then either use “zDir” or “DeconvDir” depending on which you performed last)***.

1c Drag and drop MembraneSegmentation.ijm or CytosolSegmentation.ijm from the folder “GliaMorph > AlternativeMacros” into Fiji.
2c Click “Run.”
3c Select folder with folder with input files.
4c Click “OK.”
  If you work with other data, you would need to optimize the existing segmentation workflow or produce a new one.
5c Check output: Segmented tiff stacks and folder containing MIPs.

## Basic Protocol 5 3D QUANTIFICATION OF GLIAL CELL MORPHOLOGY

Following image binarization using image segmentation, object features such as volume, surface, or thickness can be quantified. Building on this, extraction of the 3D skeleton, such as by 3D thinning (Lee, Kashyap, & Chu, 1994), allows for the quantification of skeleton length, branching points, or endpoints. Depending on the biological question, different features might be more relevant than others, and higher‐order analysis of feature selection or clustering might be insightful (Bolón‐Canedo & Remeseiro, 2020).

This step is to extract shape features from segmented images.

***For the input folder, select with segmented .tiff files —data need to be pre‐processed (i.e., subRegionTool)***.

**Figure 10:**
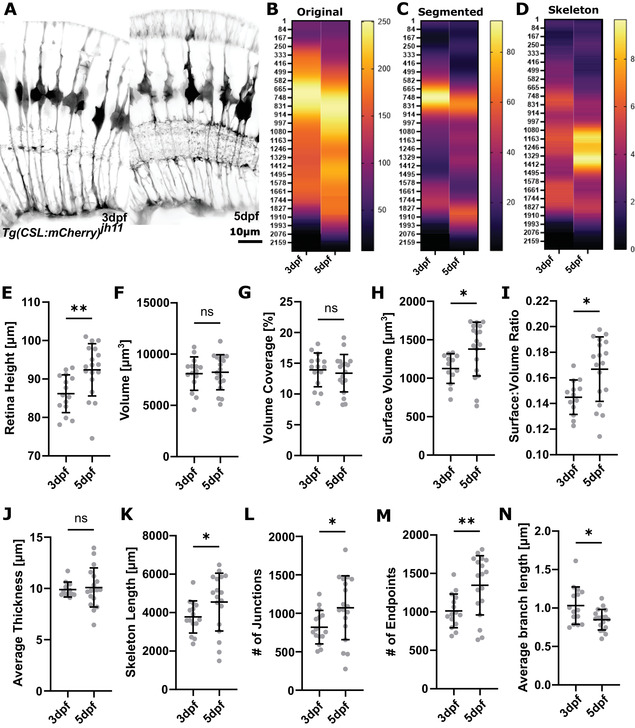
Example results. **(A)** Micrographs of representative SubregionTool output image at 3dpf and 5dpf (input for segmentation and quantification). **(B‐D)** Heatmaps showing the apicobasal texture of the original, segmented, and skeletonized image. **(E‐N)** Whole‐image quantification outputs.

1 Drag and drop step8_QuantificationTool.ijm from the folder “GliaMorph>Macros” into Fiji.
2 Click “Run.”
3 Select folder with folder with input files (depending on your above steps—select the “TH” folder you selected for Basic Protocol 4).
4 Click “OK.”
  Once the tool has finished, all windows can be closed.
5 Check Output:
  ○ Folders and files:
    ▪ **outZone folder**: Apicobasal profiles of segmented images.
    ▪ **QuantEDM**: .tiff stacks and MIPs of 3D Euclidean Distance Maps (EDM) showing local MG thickness (brighter = thicker; darker = thinner).
    ▪ **QuantSkel**: Apicobasal profiles of skeletons; skeleton .tiffs images; MIP folder (contains edges, MIP skeleton, MIP thickness).
    ▪ **Files**:
      - **QuantificationResults**: Volume [um3], PercCov [%], SurfaceVol [um3], Thickness [um].
        Use these to plot and analyze in another program.
      - **Skeleton Stats**: Contains max branch length, mean branch length, # of trees, # of branches, # of junctions, # of endpoints, # of triple points, # of quadruple points, sum of voxels.
        Use these to plot and analyze in another program.
  ○ **3D data**: Data from “QuantificationResults” and “Skeleton Stats”—these can be plotted and analyzed with other programs such as Excel or GraphPad Prism (see Table 1 for details).
  ○ **Apicobasal texture**: Plotting apicobasal (top‐to‐bottom) data distribution of original, segmented, and skeletonized data provides insights into the texture of data. Data interpretation by experts provides further insights, such as that branching is highest in the so‐called IPL or that cell bodies are located in the ONL (for example results, see Fig. 10).
***Example Data***: The folder ExampleDataProtocol5 contains input and output files.
  The quantificationTool extracts 3D skeletons using the Fiji "Skeletonize 2D/3D" Plugin (by Ignacio Arganda‐Carreras), based on 3D thinning (Lee et al., 1994) using a layer‐by‐layer removal. As this method is sensitive to small surface heterogeneities that could result in spurious branches (Attali, Boissonnat, & Edelsbrunner, 2009), users are advised to examine pre‐processing using surface smoothing or post‐processing using spurious branch pruning (Sanderson, Cohen, Henderson, & Parker, 1994).

**Table 1:** Overview of Analyzed Features With Their Variables, Units, and Description (N Indicates Embryos)

Feature	Variable	Unit	Description
Image height	I_N_	[µm]	Total height of the image
MG height	MG_N_	[µm]	Radial extension of MG
Number/count	N_N_		Number of objects in ROI
Volume	V_N_	[µm^3^]	Volume of object voxel, derived after segmentation
Volume coverage	VC_N_	[%]	Percentage of image volume covered with MG (lowest = 0; highest = 100)
Surface	S_N_	[µm^3^]	Number of object surface voxels, derived after segmentation (given in [µm^3^] for comparability between experiments)
Surface: Volume ratio	S:V_N_		Ratio of surface to volume (lowest = 0; highest = 1)
Thickness	T_N_		Distance from local centerline to the corresponding surface
Skeleton length	L_N_	[µm]	Skeleton voxels (given in [µm] for comparability between experiments)
# of Junction	J_N_		Number of points where 2/more sub‐branches branch off
# of Endpoints	EP_N_		Number of blind‐ended object points
Average branch length	BL	[µm]	Average length of individual skeleton branches

## GUIDELINES FOR UNDERSTANDING RESULTS

**Figure 11:**
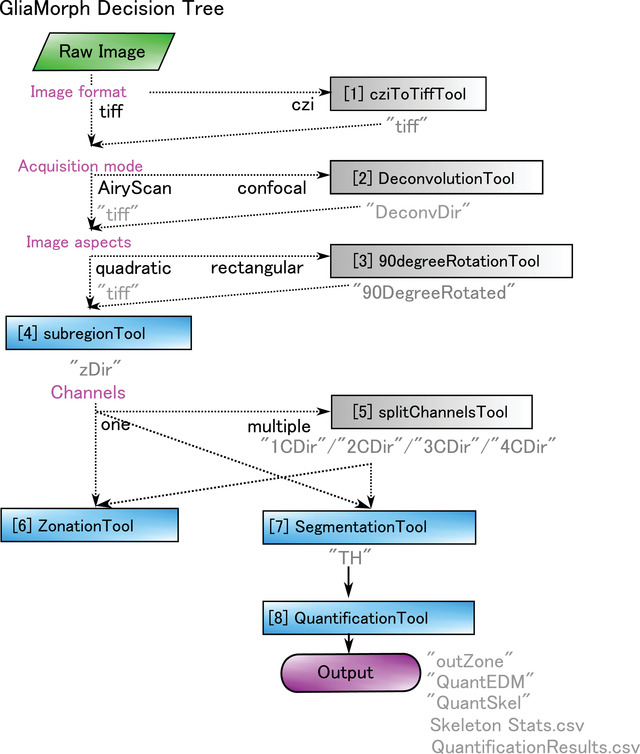
Workflow and input/output example.

GliaMorph tools are (semi‐)automated where possible. However, this means that human visual assessment is needed to check data and processing outputs for integrity. Particularly when using GliaMorph on new data, outputs and results should always be checked (workflow and folder example Fig. 11).

As most of the results will be reliant on quantification of the segmented objects, it is crucial to examine the data for over‐ and under‐segmentation. In Basic Protocol 4 we presented glia segmentation approaches and expand on it further in the section “Protocol Considerations and Adaptions” in the Commentary. Importantly, segmentation outcomes should allow for measurements of true biological values. Depending on effect sizes, inaccurate segmentation can lead to inaccurate measurements that can disguise true biological shape variations (Pincus & Theriot, 2007).

When examining quantification results in terms of the numbers produced by the individual tools rather than the image outputs, results are typically expected to be comparable within groups. This comparability can for example be assessed via the coefficient of variation (CoV), which is an indicator of variability. For example, MG of the same age and region, standardized using the SubregionTool, are typically showing less than 20% CoV. Similarly, when merging experimental repeats, data are typically expected to be homogenous and non‐clustered. Should this not be the case, we suggest studying the original data, as we experience issues like this when the embryos were not accurately staged.

The more accurate and sensitive the segmentation, the fewer samples are typically required and the smaller the effect sizes required.

As MG shape analysis is based on a linear analysis workflow, if results “seem out of place” or are “inaccurate,” we suggest users start examining the initial input data and work through the workflow step‐by‐step, as such problems are often caused by trivial issues. For example, a retina being 1200 μm in height, when expected to be in a range of 90‐110 μm, could be caused by wrong input voxel dimensions. Similarly, if no MG are found in the segmentation, the sample could have moved during the image acquisition, leading to MG being out of focus or the selected z‐stack range.

## COMMENTARY

### Background Information

#### Protocol considerations and adaptions

With this in mind, object segmentation is highly dependent on image properties, and various image segmentations exist, which have been reviewed elsewhere (Acton & Ray, 2009; Khan, 2014; Jeevitha et al., 2020). Crucially, a segmentation approach is unlikely to be directly applicable to data other than the ones it was developed for. However, while extensive segmentation validation and testing are typically applied in medical image analysis, this is often lacking in biomedical image analysis. Thus, we suggest end users dedicate an extensive amount of time to 3D data understanding, segmentation, and accompanying processing (e.g., original data: background, noise, PSF, isotropy, etc.; segmented data: connectivity, accuracy, sensitivity, etc.) before addressing feature quantification.

We cannot go into detail here on adaptions for other datasets, but suggest thorough examination of the impact of processing such as preprocessing and segmentation.

Alternatively, one could opt to use machine learning–based segmentation and feed these results into the quantificationTool.

Subsequently to segmentation, additional processing can be performed to improve segmentation results. For example, we found that removing small speckles helps to improve segmentation outcomes of antibody staining.

#### Applications of GliaMorph

GliaMorph is an image analysis toolkit that allows scientists to replace visual assessment with (semi‐) automatic objective data analysis. This is particularly useful when examining subtle phenotypes which could be easily overlooked by human visual assessment.

If wanting to apply GliaMorph to other cell types, visualization techniques, or across species, dedicated adaptions at selected steps are likely required. For example, in the case of microglia, we anticipate the main challenge to be the optimization of the segmentation, and as microglia are typically not connected, dedicated separation is unlikely to be needed. On the other hand, neuronal cells such as amacrine cells, are likely to need both adapted segmentation and separation approaches, as their properties and connectivity are highly different.

### Critical Parameters

Again, input data quality is paramount in biomedical image analysis, and a significant amount of time should be spent on understanding and optimizing data quality, particularly with data analysis requirements in mind (Wait et al., 2020). Basic Protocol 2 should inform you on (i) whether your data quality, as quantified by SNR/CNR, is sufficient for data analysis, and (ii) to which extent stacks can be meaningfully quantified (i.e., with significant z‐axis signal decay, lower z‐depth should be selected for the subregionTool).

Equally, knowledge about the data you examine will inform your analysis. For example, if the cells you examine are 1 µm in diameter, do you need a stack with 10 µm depth and 60 µm width?

This information will be particularly important for the subregionTool, as increased output image size means more information but equally increased computation time.

As mentioned above, image segmentation will be the most critical step for data quantification, and we suggest spending time and resources on this.

### Troubleshooting

Table 2 lists problems that may arise with the procedures in this article along with their possible causes and solutions.

**Table 2:** Sources and Solutions to Potential Errors

Problem	Possible cause	Solution
Macro started and immediately shows the message “Macro finished.” Without processing	Wrong input folder	Use the log file to check which input folder was selected (also, check that the correct files are in the input folder—i.e., if there are no .tiff files in it, it generally does not work).
Macro error “Stack required in line XXX […]”	MIPs selected as input instead of 3D stacks	Click okay and double‐check that the selected input folder contains the correct 3D stacks instead of MIPs (which are always indicated with “MAX_” in their name)
Memory issues		Edit › Options › Memory & Threads > change memory > restart Fiji
Step1_cziToTiffTool: “Stack is larger than 4 GB. Most TIFF readers will only open the first image. Use this information to open as raw:[…]”		Normally does not require any intervention
The output MIP folder is empty	The tool was probably run on MIPs instead of 3D stacks	Double‐check that the correct input folder was selected
Step2_DeconvolutionTool: Unrecognized command “DeconvolutionLab2 Run” in line 281	Missing plugin	Go to http://bigwww.epfl.ch/deconvolution/deconvolutionlab2/ and follow the instructions for installation
Step2_DeconvolutionTool: Unrecognized command “Diffraction PSF 3D” in line 215	Missing plugin	Go to https://www.optinav.info/Iterative‐Deconvolve‐3D.htm or https://imagej.net/Diffraction_PSF_3D and follow instructions for installation
Step2_DeconvolutionTool: Image “Final Display of RL” not found	Macro continuing to next step without finishing deconvolution	This usually happens if the images are large. Two options to solve it: 1) Increase wait time in line 282 (e.g., wait(80000)) 2) Make images smaller before running the deconvolutionTool, using the subregionTool (recommended)
Step4_subregionTool: Java.io.FileNotFoundException: FilePath\RoiSetLine.zip (the system cannot find the file specified).	RoiSetLine.zip not found.	1) RoiSetLine.zip does not exist > needs to be created by the user 2) RoiSetLine.zip exists but not in the correct folder > make sure the file is in the same folder as the input data 3) RoiSetLine.zip name is wrong > double‐check that it is correctly named/spelled (case‐sensitive!)
Step8_quantificationTool: Unrecognized command "3D Manager Options" line 61	3D ImageJ Suite Plugin missing	See Basic Protocol 1 on how to update sites
Step8_quantificationTool: Unrecognized command "Summarize Skeleton"	Neuroanatomy plugin missing	See Basic Protocol 1 on how to update sites

### Advanced Parameters

We aimed to implement the macros in such a way that no advanced parameterization is required. However, some GliaMorph tools harbor “wait functions” for which the duration can be altered, depending on data and computational setup.

### Suggestions for Further Analysis

Due to its (semi‐)automatic implementation, we anticipate that GliaMorph can be used for large‐scale data analysis, such as produced during drug screening. As GliaMorph produces quantitative objective data, MG cell phenotyping is likely to provide novel insights, particularly subtle drug‐induced cellular changes that might have been previously overlooked.

Similarly, one can link cell feature information to genomic profiles (Yuan et al., 2012) or even link feature analysis to image cytometry, which measures cellular protein and DNA in images (Tárnok, 2006).

Expanding image analysis approaches to other retinal cell types will be crucial to understanding how retinal tissue and functionality develop. Similarly, comparative studies of MG features across organisms could add to our current knowledge about MG conservation. Lastly, we believe that data integration by registration will allow the establishment of a virtual retina atlas, similar to the zebrafish brain atlas, allowing activity mapping (Randlett et al., 2015) and co‐localization analysis (Ronneberger et al., 2012). Ultimately, collating and integrating descriptive modeling data will allow the development of predictive modeling approaches.

### Author Contributions

**Elisabeth Kugler**: Conceptualization, Data curation, Formal analysis, Methodology, Project administration, Software, Validation, Visualization, Writing original draft, Writing review and editing; **Eva‐Maria Breitenbach**: Investigation, Validation, Writing review and editing; **Ryan MacDonald**: Data curation, Funding acquisition, Project administration, Resources, Supervision, Writing review and editing.

### Conflict of Interest

The authors declare no conflict of interest. Sponsors had no role in protocol design and the collection, analysis, and interpretation of data.

## Data Availability

The data that support the protocol are openly available in Zenodo at https://zenodo.org/record/5747597 [DOI: 10.5281/zenodo.5747597].
